# Correlative Stochastic Optical Reconstruction Microscopy and Electron Microscopy

**DOI:** 10.1371/journal.pone.0124581

**Published:** 2015-04-15

**Authors:** Doory Kim, Thomas J. Deerinck, Yaron M. Sigal, Hazen P. Babcock, Mark H. Ellisman, Xiaowei Zhuang

**Affiliations:** 1 Howard Hughes Medical Institute, Cambridge, Massachusetts, United States of America; 2 Department of Chemistry and Chemical Biology, Harvard University, Cambridge, Massachusetts, United States of America; 3 National Center for Microscopy and Imaging Research, Center for Research in Biological Systems, Department of Neurosciences, University of California San Diego, La Jolla, California, United States of America; 4 Center for Brain Science, Harvard University, Cambridge, Massachusetts, United States of America; 5 Department of Physics, Harvard University, Cambridge, Massachusetts, United States of America; Virginia Tech Carilion Research Institute, UNITED STATES

## Abstract

Correlative fluorescence light microscopy and electron microscopy allows the imaging of spatial distributions of specific biomolecules in the context of cellular ultrastructure. Recent development of super-resolution fluorescence microscopy allows the location of molecules to be determined with nanometer-scale spatial resolution. However, correlative super-resolution fluorescence microscopy and electron microscopy (EM) still remains challenging because the optimal specimen preparation and imaging conditions for super-resolution fluorescence microscopy and EM are often not compatible. Here, we have developed several experiment protocols for correlative stochastic optical reconstruction microscopy (STORM) and EM methods, both for un-embedded samples by applying EM-specific sample preparations after STORM imaging and for embedded and sectioned samples by optimizing the fluorescence under EM fixation, staining and embedding conditions. We demonstrated these methods using a variety of cellular targets.

## Introduction

Fluorescence light microscopy (LM) and electron microscopy (EM) are two of the most widely used imaging modalities for probing cellular structures. These modalities have distinct strengths and weaknesses that complement each other. Fluorescence LM allows spatiotemporal localization of labeled biomolecules, such as proteins and nucleic acids, with high molecular specificity and sensitivity. Multi-color imaging using spectrally distinct fluorescent labels allows several molecular targets to be imaged simultaneously and their interactions to be directly probed. Recently, various super-resolution fluorescence imaging techniques have been developed to substantially surpass the diffraction limit, allowing molecular structures in cells to be imaged with nanometer-scale resolution[1–3]. Transmission and scanning EM methods provide higher image resolution than light microscopy, including super-resolution fluorescence microscopy. Under standard staining or contrasting conditions, EM also allows, subcellular compartments, including lipid bilayer-based membranes, to be imaged with ultra-high resolution. A number of specific molecular structures can also be identified from EM images based on characteristic shapes, but such molecular structures are relatively few compared to the great diversity of macromolecules known to be made and employed to form complexes, organelles, cells and tissues. Molecule-specific imaging using EM traditionally involves immunolabeling with ferritin or gold conjugates, which contribute to low labeling efficiencies[4,5]. Several approaches have been recently developed to use genetically encoded tags, such as the tetracysteine biarsenical system[6,7], miniSOG[8] and APEX2[9,10], to provide EM contrast for specific molecules. These approaches give much higher labeling efficiency than immunogold. However, “multi-color” EM imaging of more than one or two molecular targets still remains challenging. It is thus desirable to perform correlative fluorescence LM and EM such that the spatial locations of specific molecules can be determined using fluorescence imaging in the context of the cellular ultrastructure revealed by EM.

Correlative fluorescence LM and EM has been reported for both conventional[11–14] and super-resolution fluorescence imaging[15–25]. In particular, correlative super-resolution LM and EM methods have been reported for unembedded samples using negative staining[15,17,19,22,25] or platinum replica for EM contrast[18,21], in plastic-embedded samples using negative staining for EM contrast[16,20,23], and in cryoEM samples without any metal staining[24]. Although these studies have demonstrated the impressive power of correlative microscopy, it remains challenging to obtain optimal quality for both EM and super-resolution LM images in correlative microscopy. For example, the light emission from fluorescent proteins and dyes can be substantially compromised in the acidic and oxidizing conditions typically used for plastic embedding protocols. Osmium tetroxide, which is often used as a strong fixative and stain for membrane structures in EM, can severely quench the fluorescence signal and hence an extremely low concentration of osmium tetroxide (0.001% OsO_4_ and 0.1% KMnO_4_) has been used to minimize the photon loss of fluorescent proteins in correlative super-resolution imaging[16,20]. A more recent study uses organic fluorophores for super-resolution imaging and another weak fixative and stain, uranyl acetate, for EM contrast[23]. These relatively mild fixatives and stains compromise the ultrastructure preservation and membrane contrast in the EM images. Both studies use the acrylic resin as the embedding material[16,20,23], whereas the harder epoxy-based resins are known to be better for ultra-structure preservation and high-quality ultrathin sectioning[16]. Moreover, since photoswitching of the dye molecules required for super-resolution imaging is partially inhibited when the dyes are embedded in resins, the quality of super-resolution images is also compromised by the resin embedment[23]. Correlative super-resolution fluorescence and SEM/platinum-replica TEM studies[18,21,22,25] circumvent the above problems by performing fluorescence imaging before staining for EM, but these approaches can only be applied to imaging structures relatively close to the cell/sample surface. Correlative imaging with cryo-electron tomography[24] does not require any fixation or staining for EM contrast, but the long-working distance objective (NA ≈ 0.7) required for imaging samples in the cryo chamber has a relatively low photon collection efficiency, giving lower fluorescence resolution. Thus, additional method development is needed to complement the above approaches and optimize the power of correlative super-resolution fluorescence and electron microscopy.

Here, we present several correlative super-resolution fluorescence and electron microscopy assays by combining three-dimensional (3D) stochastic optical reconstruction microscopy (STORM)[2,26,27] with several EM imaging modes. These include protocols for imaging unembedded samples and protocols for imaging embedded and sectioned samples. In the former case, we performed all EM-related sample treatments, which could affect the brightness and photoswitching properties of dyes, after STORM imaging, hence both STORM and EM imaging conditions could be separately optimized. For the embedded and sectioned samples, we compared various embedding materials and conditions, fixatives, and negative staining agents to find near optimal conditions for both STORM and EM imaging. We applied these protocols to several cellular and viral structures.

## Results

### Correlative 3D STORM and transmission EM (TEM) or scanning EM (SEM) for un-sectioned samples

We first present a simple correlative 3D STORM and TEM imaging method for unembedded samples. In this method, most of sample preparations for either STORM or TEM were not changed from the standard protocols used for each imaging modality. STORM imaging was performed prior to any sample preparation required for EM imaging. Therefore, as the fluorescence signal acquisition precedes the addition of any EM fixative or stains, the optimal fixative and stain could be used for EM, and hence neither STORM nor EM images were substantially compromised.

**Fig 1:**
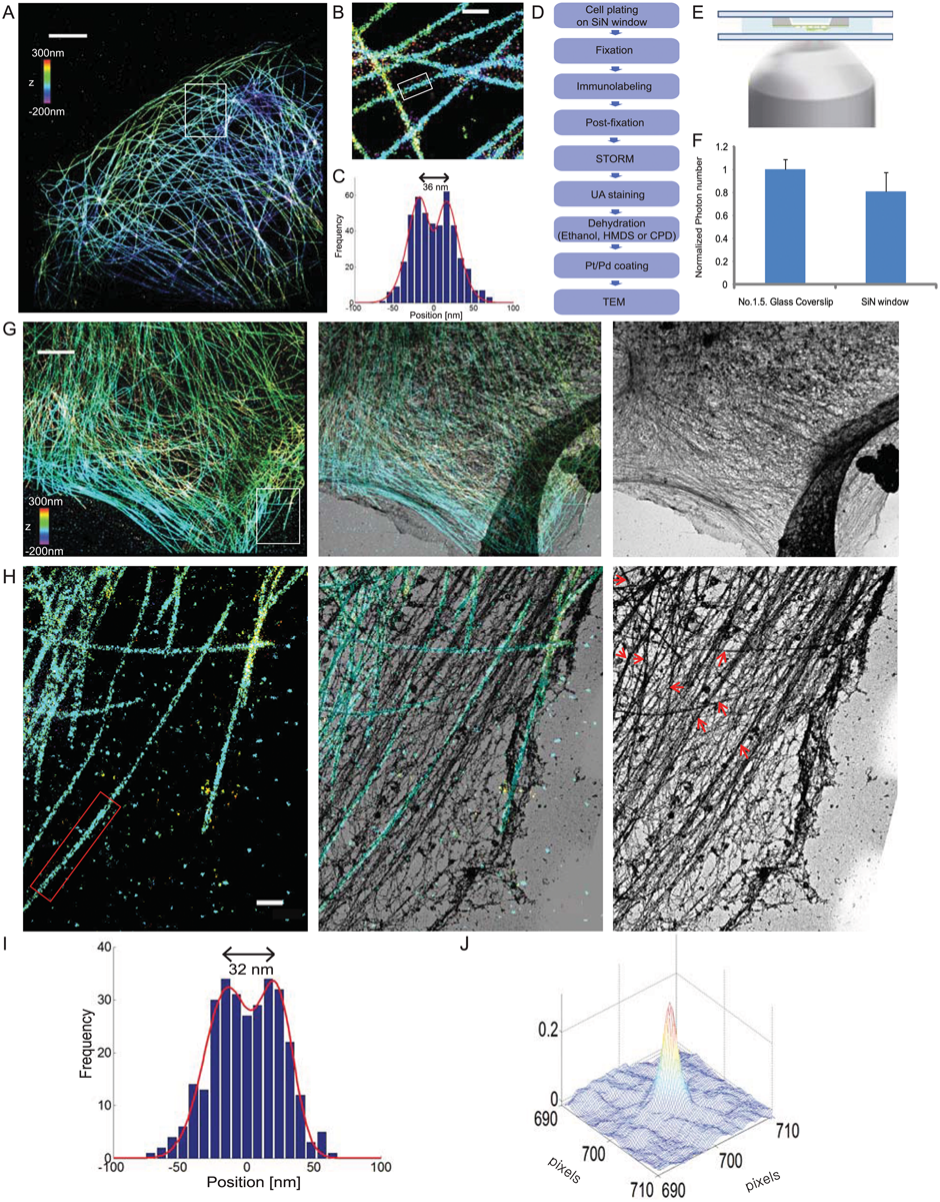
Correlative 3D STORM and TEM images of immunolabeled microtubules in a BS-C-1 cell. (A) 3D STORM image of microtubules in a BS-C-1 cell on a No. 1.5 glass coverslip. (B) Magnified views of the boxed regions in (A). (C) Transverse profiles of localizations corresponding to regions boxed in white in b. Blue bars: localization frequency measured from the STORM image. Red line: Gaussian fit of the blue bars. (D) Flowchart of the major steps in correlative 3D STORM and TEM imaging of unembedded samples. (E) Scheme of the SiN window and the sample mounting geometry. (F) Normalized number of photons detected on the samples mounted on the normal No. 1.5 coverslip and the SiN window. The results are normalized to the average photon number obtained from samples on the No. 1.5 glass coverslip (photon number = 5233). (G) Correlative 3D STORM and TEM images of microtubules in a BS-C-1 cell. Left: STORM image. Right: TEM image. Middle: Overlaid image. (H) Magnified views of the boxed regions in (G). Red arrows in TEM image: thick filaments corresponding to microtubules. (I) Transverse profiles of localizations corresponding to regions boxed in white in (G). Blue bars: localization frequency measured from the STORM image. Red line: Gaussian fit of the blue bars. (J) Cross-correlation between STORM and TEM images. Scale bars, 5 μm in (A,G) and 500 nm in (B,H).

To image the same sample by both STORM and TEM, we used silicon nitride support films (SiN window), which are transparent to both visible light and electron beams. We chose microtubules in cells as our first target to test this imaging approach. We first performed 3D STORM imaging of the microtubules in cells plated on No. 1.5 coverslips to check for the STORM image quality without performing any EM imaging (Fig 1A–1C). BS-C-1 cells were washed in PBS, and fixed and permeabilized using 0.3% glutaraldehyde and 0.25% Triton X-100 in cytoskeleton buffer (CB: 10 mM MES pH 6.1, 150 mM NaCl, 5 mM EGTA, 5 mM glucose, and 5 mM MgCl_2_) for 5 min in the first step, followed by the second fixation step using 2% glutaraldehyde in CB for 15 min. We then performed immuno-labeling of microtubules with tubulin antibodies and Alexa 405 and Alexa 647 co-labeled secondary antibodies. Here, Alexa 647 serves as the photoswitchable dye and Alexa 405 facilitates the activation of Alexa 647 by low power 405 nm light[28]. Individual microtubule filaments were clearly resolved by STORM (Fig 1A and 1B). The hollow tubular shape of the immuno-stained microtubules was resolved in the transverse profile, which showed two peaks separated by 36 nm in agreement with the previous reported values[29].

For correlative 3D STORM and TEM, the sample was prepared in the same way, but on the SiN window (Fig 1D and 1E). The SiN window was sandwiched between two No. 1.5 coverslips for imaging, as shown in Fig 1E. In this imaging geometry, because the cell sample is at a distance from the coverslip, we had to consider the refractive index mismatch between the imaging buffer and the coverslip, which could cause substantial spherical aberration in STORM imaging[30]. To alleviate this effect, we used an index-matching imaging medium, which contains 60% sucrose and 5% glucose (refractive index ~1.45)[30]. The brightness of the Alexa 647 dye (measured as the number of photons detected per switching event) was reduced by ~19% in this geometry compared to the case where the cell is directly plated on the No. 1.5 coverslip (Fig 1F). The 3D STORM image could still resolve hollow tubular shape of the microtubules, with 32 nm width (Fig 1G–1I). After 3D STORM imaging, the sample was stained with 0.1% aqueous tannic acid and 0.2% uranyl acetate, dehydrated with graded ethanol steps, and coated with a Pt/Pd layer ~ 1nm thick, followed by TEM imaging (Fig 1D). We correlated the 3D STORM image to the TEM image using SiN mesh in the SiN window as fudicial markers. All of the microtubules observed in the 3D STORM image were also observed in the TEM image (Fig 1G and 1H) and all of the thick filaments, likely representing microtubule structures in the TEM image, were found in the STORM image(Fig 1H right panel, examples marked by red arrows). Other types of cytoskeletal elements that did not appear in the STORM image, typically appearing as thinner filaments compared to microtubules, could also be identified in the TEM image, presumably corresponding to actin and intermediate filaments. In order to estimate how accurate the correlation is, we calculated the normalized cross-correlation between STORM and TEM images and displayed it as a surface plot (Fig 1J). A sharp peak in the correlation map is clearly observed at the center and a simple Gaussian fit to the profile yielded a displacement of 1.3 nm from the center and a FWHM of 44.5 nm. The displacement of the peak from the center indicates the alignment errors between the STORM and EM images and the width of the peak is largely dominated by the feature sizes in the images.

**Fig 2:**
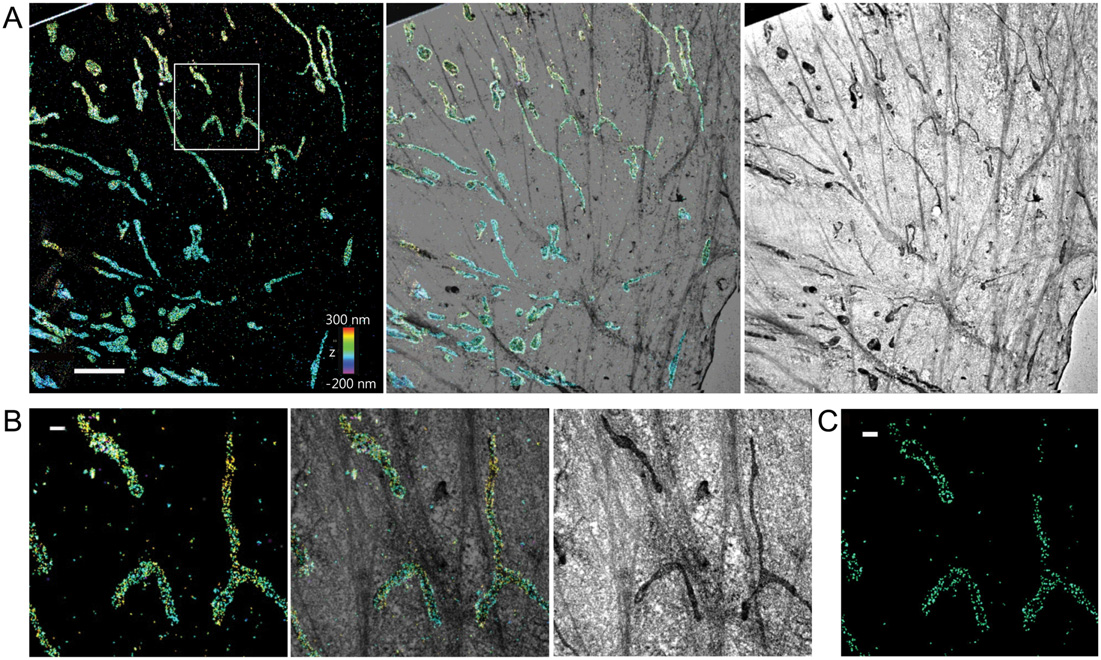
Correlative 3D STORM and TEM images of immunolabeled mitochondria in a BS-C-1 cell. (A) Correlative 3D STORM and EM images of mitochondria in a BS-C-1 cell innumo-stained for TOM20. Left: STORM image. Right: SEM image. Middle: Overlaid image. Part of SiN mesh is visible on the upper left corner of the image. (B) Magnified views of the boxed regions in A. (C) xy cross-section of the 3D STORM image of mitochondria in the same region. Scale bars, 5 μm in (A) and 500 nm in (B, C).

Next, we imaged mitochondria in cells with correlative 3D STORM and TEM (Fig 2). The cells were again plated on the SiN window and assembled in the imaging chamber as described above. We used standard indirect immunological fluorescence methods to stain the outer membrane of mitochondria in BS-C-1 cells, as described previously[30]. After fixation with 4% PFA, we stained Tom20, an outer mitochondrial membrane protein, using primary antibodies followed by Alexa 405 and Alexa 647 co-labeled secondary antibodies. We could observe the expected hollow shape of the mitochondria in the 3D STORM image and individual mitochondria correlated with their counterpart in the TEM image (Fig 2).

For target structure that is near the sample surface or exposed, SEM offers a simpler alternative [22,31]. For correlative STORM and SEM imaging, here we used commercial photo-etched gridded coverslips (No.2 thickness) to make it easy to find the same regions of interest (ROI) in the STORM and EM setups. As a test of this correlative imaging approach, we imaged filamentous influenza viruses budding from infected cells. To prepare the sample, we infected human lung epithelial adenocarcinoma A549 cells with a filamentous virus strain (Udorn) for 12 hours and fixed the infected cells with 4% paraformaldehyde (PFA) and 0.1% glutaraldehyde (GA), followed by indirect immuno-labeling of the viral envelope protein HA with primary antibodies and Alexa Fluor 405 and Alexa Fluor 647 co-labeled secondary antibodies.

**Fig 3:**
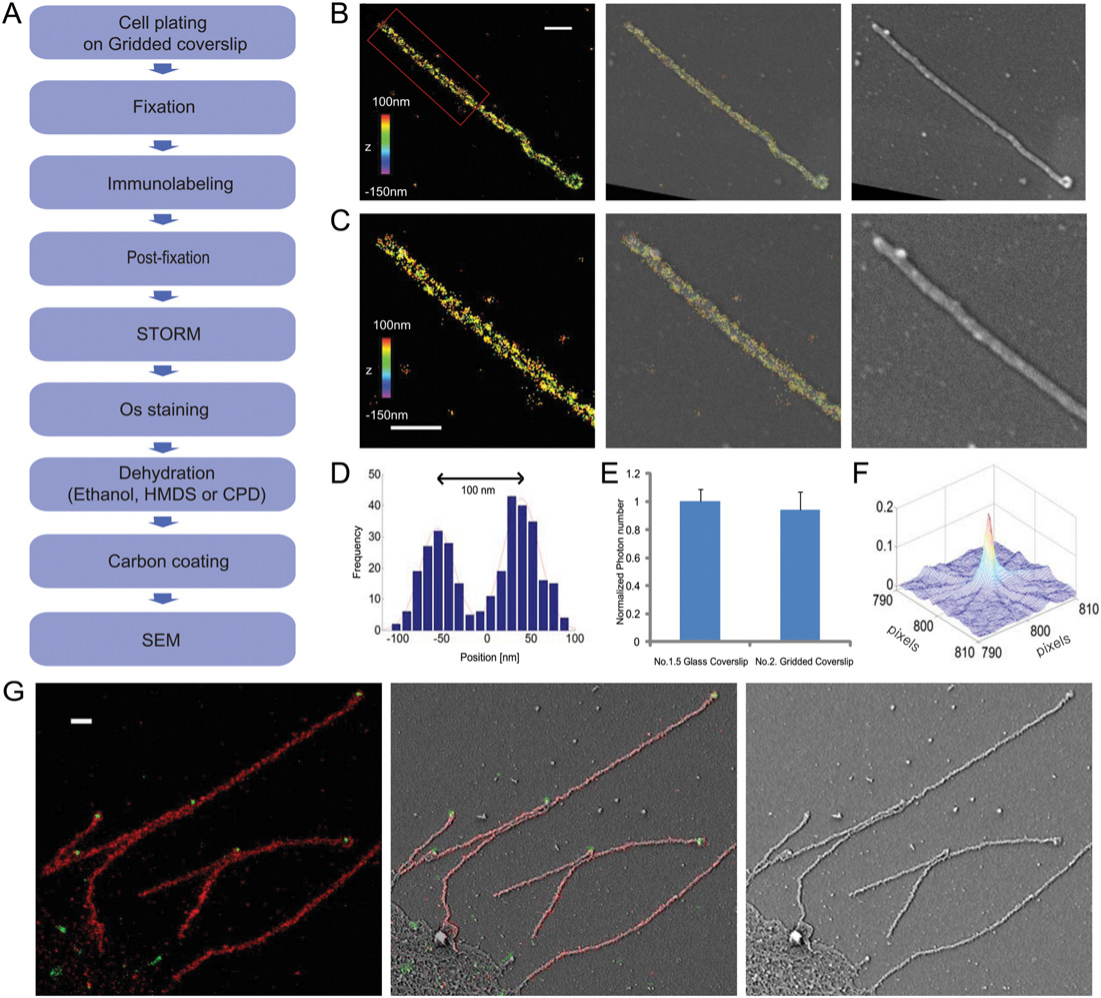
Correlative 3D STORM and SEM imaging of individual filamentous influenza viruses. (A) Flowchart of the major steps in the correlative 3D STORM and SEM imaging of unembedded samples. (B) Correlative 3D STORM and SEM images of Udorn virus immuno-labeled for HA. Left: STORM image. Right: SEM image. Middle: Overlaid image. (C) Magnified views of the boxed regions in (B). (D) Transverse profiles of localizations corresponding to regions boxed in red in (B). Blue bars: localization frequency measured from the STORM image. Red line: Gaussian fit of the blue bars. (E) Normalized photon numbers per switching event. The results are normalized to the average photon number obtained from samples on the No. 1.5 glass coverslip (photon number = 5233). (F) Cross-correlation between STORM and SEM images. (G) Correlative two-color STORM and SEM images of budding Udorn virus filaments immuno-labeled for M1 (red) and vRNP (green). Left: STORM image. Right: SEM image. Middle: Overlaid image. Scale bars, 500 nm in (B,C and G).

We first obtained 3D STORM images of the virus filaments on the photo-etched gridded No. 2 coverslips (Fig 3A). The 3D STORM image and the xy cross-section show that we could resolve hollow shape of the virus filament (Fig 3B–3D). Analysis of the xy cross-section revealed a filament width of 100 nm in the STORM image, which agrees with the known diameter of a virus filament (80–120 nm)[32]. The number of photons detected per switching event of Alexa 647 decreased only slightly (by 6%) owing to the use of the thicker photo-etched gridded coverslip as compared to the No. 1.5 coverslip (Fig 3E). After STORM imaging, the sample was stained with 1% osmium tetroxide, dehydrated in a graded ethanol series, dried by either CO_2_ critical point drying or transition to hexamethyldisilazane, coated with carbon, and imaged in an SEM using the secondary electron detector imaging mode. The SEM images correlated well with the STORM images with viral filament width measured to be 85nm (FWHM) in the SEM image. The cross-correlation map between the STORM and SEM images (Fig 3F) clearly shows a sharp peak at the center (displacement error of 3.3 nm and FWHM of 33.7 nm).

We also performed correlative two-color STORM and EM of filamentous virus immuno-stained for both the viral matrix protein M1 and viral ribonucleoproteins (vRNPs) (Fig 3G). Again, we infected A549 cells with filamentous Udorn virus for 12 hours and fixed with 4% PFA and 0.1% GA, followed by immuno-labeling with M1 and vRNP antibodies, and Alexa 647-labeled (for M1) or Alexa 568-labeled (for vRNP) secondary antibodies, respectively. Correlative images showed that the majority of budding viruses have vRNPs located at the distal end, consistent with a previous cryo-EM observation [33].

### Correlative STORM and back scattered electron (BSE)-SEM imaging of embedded and sectioned samples

To probe the interior regions of thick biological samples using EM, the samples are typically embedded in resins and thin-sectioned using an ultramicrotome. To this end, we developed a correlative STORM and BSE-SEM imaging assay for resin-embedded and sectioned samples. We chose BSE-SEM as the EM imaging mode because it could be applied to samples mounted on glass coverslips, which is convenient for STORM imaging. It is also straightforward to extend the protocol describe here to correlative STORM and TEM imaging by using TEM grids instead of coverslips for mounting samples.

The major differences between this method and the methods describe earlier are two fold: 1) the embedding procedure and choice of embedding resins could affect the brightness and photoswitching properties of the fluorophores used for STORM imaging as well as the ultrastructural preservation, and 2) EM-related fixatives and stains need to be applied to the sample prior to embedding and sectioning, and hence could also affect the properties of the fluorophores. To minimize the effects of these factors on the STORM and EM image quality, we tested various types of resins, polymerization strategies, EM fixatives and stains, and exploit a chemical etching approach to expose the epoxy-embedded dye to the imaging buffer to optimize photoswitching.

STORM imaging relies on the photoswitching and localization of individual fluorophores. For many dye molecules, such as Alexa 647, the switching performance can be enhanced by a primary thiol in the imaging buffer[34]. Alexa 647 switched relatively poorly in resin embedded sections, likely because the plastic resin blocked the access of the thiol to the dye molecules. We therefore adapted an approach to expose the dyes in the embedded and sectioned sample to the imaging buffer by chemical etching the sections with sodium ethoxide, which has previously been used to enhance immuno-gold labeling[35]. This etching procedure substantially improved the switching performance of dye molecules. In the following examples, all fluorophore brightness measurements and STORM imaging of embedded and section samples were performed after etching.

**Fig 4:**
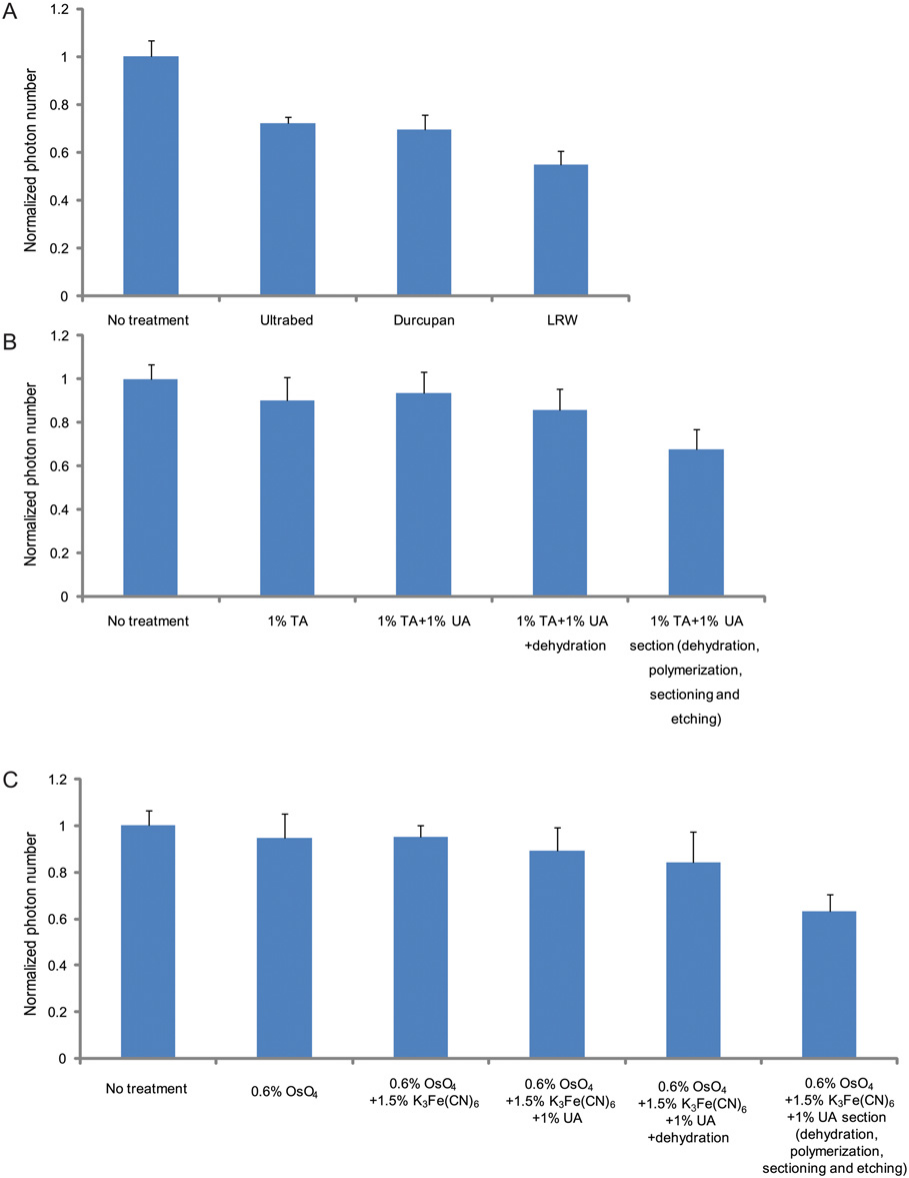
The average photon numbers per switching event of dye molecules under various conditions used in the correlative STORM and EM imaging. (A) Normalized photon numbers in different resins. (B) Normalized photon numbers detected after various steps of sample treatment in the case of tannic acid—uranyl acetate fixed, Ultrabed-embedded samples. (C) Normalized photon numbers detected after various steps of sample treatment in the case of the osmium—potassium ferricyanide—uranyl acetate fixed, Ultrabed-embedded samples. The photon numbers in all cases are normalized to the control sample (photon number = 5233), which was post-fixed only with 4% PFA and 0.1% GA after immunolabeling without any additional membrane fixation (by osmium, tannic acid or uranyl acetate), dehydration, polymerization, sectioning or etching.

For embedding resins, we tested two epoxy-type resins, Ultrabed (Spurr-like) and DurcupanACM as well as an acylic-type resin, LR White. The acrylic resin is preferred for post-embedding immono-labeling because the relatively porous structure of the acrylic resin partially preserves the antigenicity and allows antibodies greater penetration and access to epitopes[36]. On the other hand, the epoxy resins are generally considered better for ultrastructural preservation and for higher quality sectioning. Even though the acrylic resin partially preserves the antigenicity, the antibody labeling density was substantially lower than that obtained on unembedded samples and hence limited the resolution of the STORM images. We thus chose to immunolabel the samples prior to embedding in resins in this work. We measured the brightness (i.e. the number of photons per switching event) of the fluorescent dyes embedded in Ultrabed, Durcupan and LR White. Because the UV light-curing procedure typically used for curing LR White severely quenched the fluorescence, we used a chemical curing method instead of UV light. The measurement results (Fig 4A) indicate that the fluorescence signals of dyes are better preserved in the epoxy resins, Ultrabed and Durcupan, than in the acrylic resin LR white. Consequently, in the following experiments, we use Ultrabed as the embedding resin.

For resin-embedded samples, a strong fixative is essential to cross-link cellular structures and protect the tissue from major distortions that can be caused by the dehydration and resin embedding procedures. This is especially true for preserving membrane structures[37]. Many fixatives, such as osmium tetroxide and uranyl acetate both fix and stain. Others like tannic acid do not stain but instead act as a mordant to enhance binding of metals to increase contrast in EM images. However, strong fixation conditions and some metals can quench fluorescence[38]. For example, osmium tetroxide strongly quenches signals from fluorophores because it is a very strong oxidizing agent[39] and it has been shown previously that signals from fluorescent proteins are reduced by more than 90% even with a very low concentration (0.1%) of osmium tetroxide[16]. To obtain high-quality correlative STORM and EM images, we tested both an osmium free EM fixative/stain, containing a tannic acid (1%)—uranyl acetate (1%) combination and a non-oxidative osmium [osmium tetroxide (0.6%) and potassium ferricyanide (1.5%)]—uranyl acetate (1%) combination. Tannic acid was used in the first case in combination with uranyl acetate as uranyl acetate alone is not sufficient to generate high EM contrast of membranes[23,37]. The concentrations were chosen such that the fluorescent signals of the dyes used here were not substantially quenched by the fixative/stain agents. As the acidic pH conditions typically accompanying these fixatives also quench the fluorophore signal, we thus adjusted pH of fixatives to maximize the fluorescence signal[39]. For example, when 1% aqueous tannic acid solution and 1% aqueous uranyl acetate solution (~pH 4) was used, the fluorescence intensity of the Alexa 647 dye was reduced by 35%, whereas, when the pH of the tannic acid and uranyl acetate solution was adjusted to 8.0 using Tris buffer, the fluorescence reduction was only 7%. Similar results were observed for the non-oxidative fixatives.

**Fig 5:**
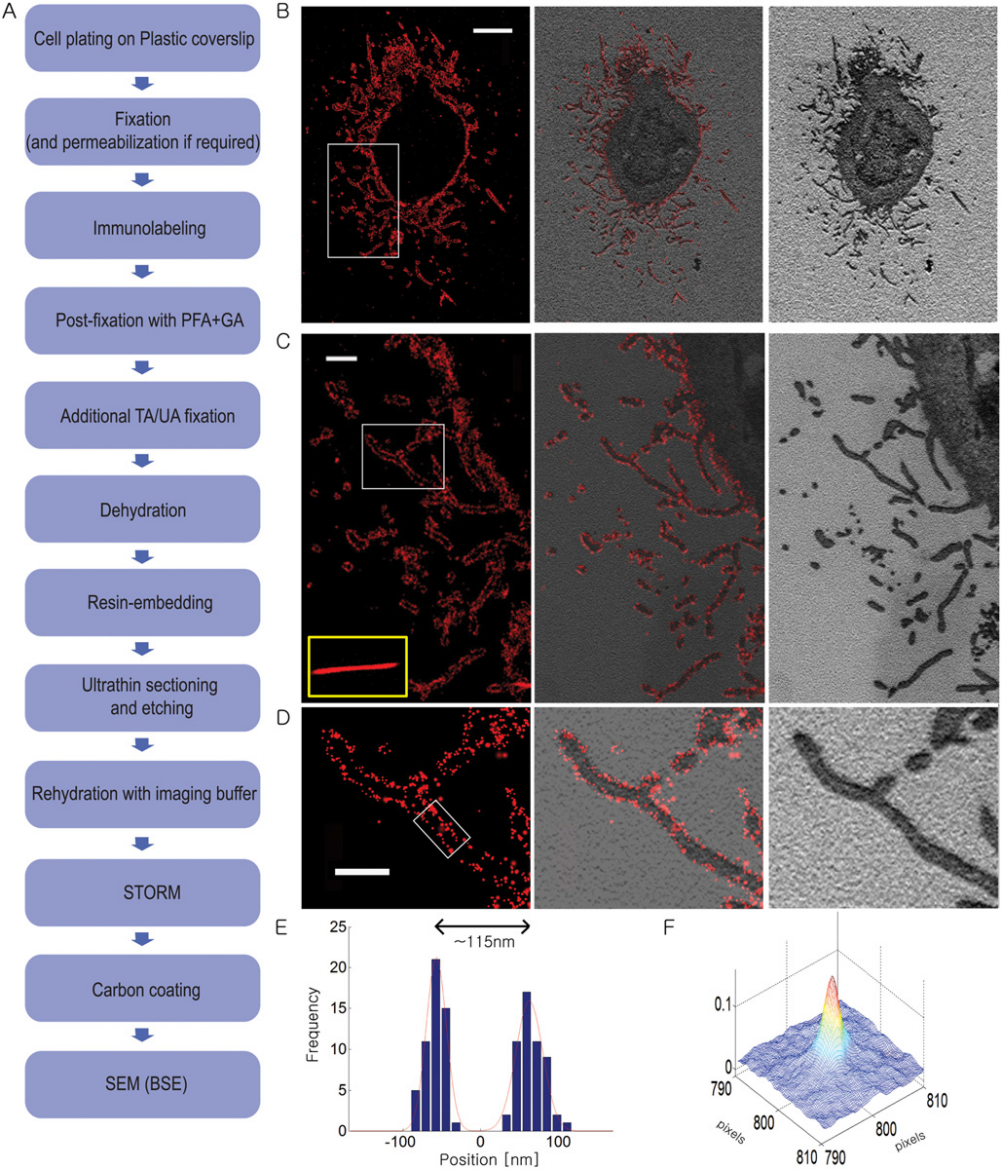
Correlative STORM and SEM-BSE images of resin-embeded sections containing filamentous influenza viruses budding from infected cells. (A) Flowchart of the major steps in correlative 3D STORM and BSE-SEM imaging of embedded samples. (B) Correlative STORM and EM images of influenza infected A549 cells immuno-stained for HA. Left: STORM image. Right: SEM image. Middle: Overlaid image. (C) Magnified views of the boxed regions in a (B). Inset in yellow box: STORM image of an influenza virus filament, immuno-stained for HA, embedded in Ultrabed section without etching. (D) Magnified views of the boxed regions in (D). (E) Transverse profiles of localizations corresponding to regions in the white box in (D). Blue bars: localization frequency measured from the STORM image. Red line: Gaussian fit of the blue bars. (F) Cross-correlation between STORM and BSE-SEM images. Scale bars, 5 μm in (B) and 500 nm in (C, D).

As the first test of the correlative STORM and BSE-SEM method, we again imaged filamentous influenza virus budding from cells. We plated the cells on plastic coverslips as these coverslips can be relatively easily removed from the hardened resin block after polymerization. After virus inoculation, the infected cells were fixed by 4% PFA and 0.1% GA and immuno-labeled with anti-HA primary antibody and Alexa 647-labeled secondary antibody. The labeled sample was then post-fixed with 4% paraformaldehyde and 0.1% glutaraldehyde to covalently attach the immuno-label to the sample to minimize loss during dehydration and embedding. We used tannic acid—uranyl acetate to further fix and stain the sample so as to enhance the membrane contrast for EM imaging. We then dehydrated the fixed sample, embedded the sample in Ultrabed resin, and sectioned the sample at a thickness of ~70nm. Finally, we etched the sections with sodium ethoxide, dried the sections on the hot plate at 60°C and rehydrated with imaging buffer for STORM and then BSE-SEM imaging (Fig 5A). The STORM and EM images are well correlated (Fig 5B–5D), and show the hollow tubular structures of viral filaments with expected width (Fig 5E). The corresponding BSE-SEM image (Fig 5C and 5D) also provides decent contrast at the membrane boundaries. The cross-correlation between the STORM and EM images (Fig 5F) again appeared as a sharp peak close to the center, implying a high correlation between two images (displacement error of 2.5nm and FWHM of 45.9nm).

**Fig 6:**
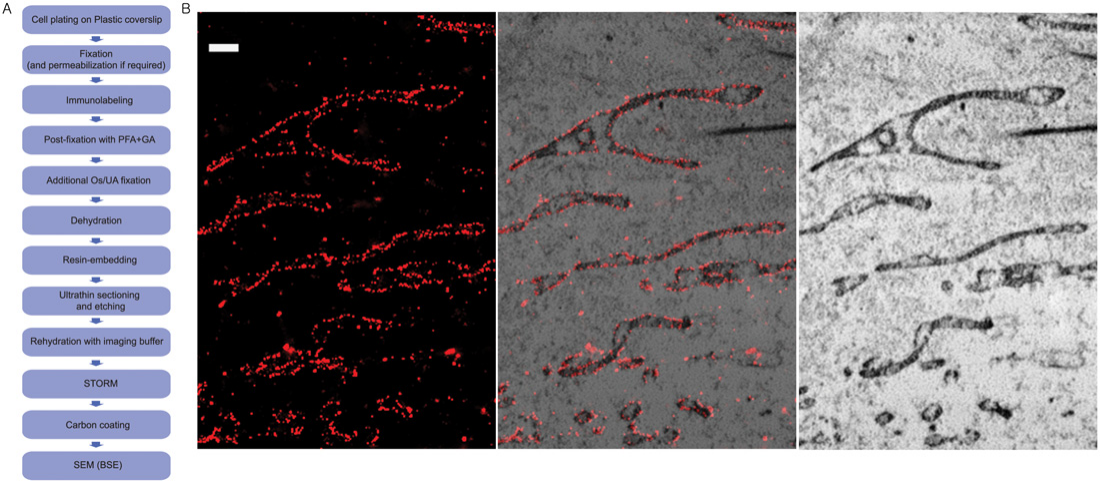
Correlative STORM and BSE-SEM images of immunolabeled mitochondria in a resin-embeded sections of a BS-C-1 cell. (A) Flowchart of the major steps in correlative 3D STORM and BSE-SEM imaging of embedded samples. (B) Correlative 3D STORM and EM images of cells immuno-stained for TOM20. Left: STORM image. Right: SEM image. Middle: Overlaid image. Scale bars, 500 nm.

Next, we performed correlative STORM and BSE-SEM imaging of an intracellular membrane-bound organelle, the mitochondria. After fixation with 4% PFA and 0.1% GA, BS-C-1 cells were permeabilized with 0.2% Triton and immuno-stained for the mitochondria outer membrane protein Tom20 with primary antibodies and secondary antibodies doubly labeled with Alexa 405 and Alexa 647. The immuno-labeled cells were post-fixed with 4% PFA and 0.1% GA before further membrane fixation, staining, embedding and sectioning followed by STORM and then EM imaging (Fig 6A). Because the EM contrast of intracellular membranes tends to be low after cell permeabilization, we used the stronger membrane fixative/stain, the non-oxidating osmium-uranyl acetate combination, here to enhance the membrane contrast of mitochondria. The correlated STORM and BSE-SEM images of mitochondria are shown in Fig 6B, with the STORM images of TOM20 showing the expected outer membrane staining and the EM images show the typical cristae structure of mitochondria.

Since the localization precision of individual fluorophores and hence the STORM image resolution depend on the number of photons detected per switching cycle of the dye[29,40], we quantitatively characterized the effect of each sample preparation steps on the brightness of the fluorophores, i.e. the number of photons detected per switching event of Alexa 647 (Fig 4B and 4C). Fig 4B shows the fluorescence signal changes in the case where the tannic acid—uranyl acetate combination was used as the membrane fixative and stain. The tannic acid and uranyl acetate addition cause a 6% decrease in the photon number, and the following dehydration step induced another 8% decrease. Finally the resin embedding, sectioning and sodium ethoxide etching steps caused a further 18% reduction in the photon number, in which the resin embedding made the largest contribution (Fig 4B). Thus, photon number was overall reduced to 68% of the original value of Alexa 647 for the embedded and sectioned sample in this case. In the case where the osmium—potassium ferricyanide—uranyl acetate combination was used as the membrane fixative and stain, photon reduction was similar: 11% reduction by osmium—potassium ferricyanide—uranyl acetate, 5% by dehydration, and 20% by embedding, sectioning and etching. Overall, the fluorescence signal was reduced to 63% of the original value. We note that although a higher concentration of the membrane fixatives/stains would increase the EM contrast, it would lead to larger decrease in the brightness of the fluorophores. Interestingly, compared to photoactivatable fluorescent proteins, in which the fluorescence signal was reduced by more than 90% with only 0.1% of osmium tetroxide[16], photoswitchable dyes appeared to be much more robust when exposed to membrane fixatives/stains, leading to less of a compromise between STORM and EM imaging. We also quantitatively characterized the effect of each sample preparation steps on the number of switching cycles per fluorophore. In contrast to the photon number, the number of switching cycles did not substantially change after various steps of sample treatment from the control sample. In the control sample prior to any EM-related sample treatment, the Alexa 647 flurophore switches 14.3 times on average before bleaching. After the various EM-related sample preparation steps (treatment with EM fixatives/stains, dehydration, and embedding), the average numbers of switching cycles per fluorophore are 13.6–14.3.

## Discussion

Here, we report several correlative STORM and EM assays for imaging both unembedded samples and resin-embedded, sectioned samples. For the unembedded samples, the best results were obtained by doing the EM-specific sample treatment, such as membrane fixation and negative staining, dehydration, and metal/carbon coating after STORM imaging. In this way, the conditions for STORM and EM imaging could be separately optimized. However, since multiple sample treatment steps occur between STORM and EM imaging, it is important to minimize the extraction or distortion of samples between these two imaging steps to produce highly correlated images. If the samples were not fixed sufficiently or if the dehydration procedure was too harsh or too long, the cellular ultrastructure could be degraded between STORM imaging and subsequent processing for EM imaging.

For the embedded samples, because EM-specific fixation and staining have to be applied to the sample prior to the resin-embedding step and STORM imaging is performed after embedding and sectioning, it is important to optimize the fixation/staining conditions and embedding resin materials such that the emission property of the fluorophores used for STORM imaging is not substantially altered, while still providing sufficient preservation and contrast to observe cellular ultrastructure in the EM image. Strong fixatives/stains could quench emission from fluorophores but weak fixatives/stains will not preserve ultrastructure or provide sufficient EM contrast. Compared to previous studies[16,20,23], we used stronger fixatives/stains to preserve cellular ultrastructure and enhance membrane contrast in EM. We found that the fluorescence signal from the dyes were largely preserved under these strong fixation and staining conditions. Interestingly, organic dyes appeared to be much less perturbed by the fixatives than fluorescent proteins. The choices of embedding resins can also affect fluorescence emission and ultrastructure preservation. Instead of using acrylic resins [16,20,23], we used epoxy resins here because they are known to be better for ultrastructural preservation and sectioning. We also found the fluorescence signals of dye molecules to be better preserved in epoxy resins than in acrylic resins. Finally, because resin embedding can prevent the switching agents from reaching the embedded dyes and compromise the photoswitching property of the dyes, we also applied a chemical etching step to partially remove the resin material after sectioning and recover the photoswitching behavior of the dyes. Without this etching step, the switching of the dyes was substantially inhibited and the STORM image quality was substantially degraded. For example, when imaging filamentous influenza virus in embedded sections without etching, we found it difficult to switch off the Alexa 647 dye molecules in order to reach single-molecule imaging conditions. Often more than one dye molecule was on per diffraction-limited area, making it difficult to localize these molecules precisely. As a result, we were not able to resolve the hollow tubular shape of the viral envelope (Fig 5C, inset in the left panel).

We note that the assays reported here are complementary to, but not a replacement of, previously developed correlative super-resolution fluorescence and electron microscopy methods. For example, the use of a platinum replica could provide substantially higher contrast for some cellular structures than negative stains[18,21,22,31,41], but can only be used to image structures near the surface of a specimen. For samples labeled with fluorescent proteins instead of dyes, embedding in epoxy resins largely quenches the fluorescence signal and acrylic resins need to be used[16]. Preservation of the signal of fluorescence proteins also requires weaker membrane fixatives/stains than what we used here[16]. Fluorescent dyes and proteins have distinct advantages and disadvantages in labeling biological samples—dyes are substantially brighter than fluorescent proteins and also allow endogenous proteins and nucleic acids to be labeled, but dye labeling is more difficult for living cells and also generates higher non-specific background than genetic fusion with fluorescent proteins. Genetic labeling with both fluorescent proteins and dyes (through a protein/peptide tag) allows correlative super-resolution fluorescence and cryo-EM imaging[24]. This correlative imaging mode has the advantage of avoiding chemical fixation and better preserving the native state of cellular structures, but the fluorescence image resolution is compromised due to the imaging geometry required for cryogenic samples. Future work is required to further explore the power of correlative imaging, for example, through the development of correlative live super-resolution fluorescence imaging and EM, either through fast fixation or freezing after fluorescence imaging or through liquid-chamber EM imaging[42,43]. We anticipate that correlative super-resolution fluorescence and electron microscopy will be a valuable tool for ultrastructural studies of many cellular processes.

## Methods

### Substrates cleaning

Various substrates were used depending on the experiments. For 3D STORM imaging without EM imaging, eight-well coverglass chambers (Labtek, 154534; Nunc) were used. Photo-etched gridded coverslips (Electron Microscopy Sciences, 72264–23) were used for correlative 3D STORM and SEM of hydrated, unembedded samples. For correlative 3D STORM and TEM of unembedded samples, silicon nitride support film (Ted Pella, 21515–10) was used. For correlative STORM and BSE-SEM imaging embedded, sectioned samples, plastic coverslips (Thermanox, 174950) were used for cell culture, which allow easy detachment of coverslips from the hardened resin block after polymerization, and #1.5 glass coverslips (Electron Microscopy Sciences, 72204–04) were used to mount the sections for STORM imaging. All of the substrates described above were cleaned by sonication for 20 min in 1 M aqueous potassium hydroxide and the washed thoroughly with MilliQ water, except for the silicon nitride support films, which were washed briefly with 1 M aqueous potassium hydroxide and MilliQ water without sonication because these thin films (40–200 nm thick) are fragile. The cleaned glass coverslips used for supporting the embedded sections were additionally glow-discharged using a Glow discharge system (Agar Sceintific AGB8960) to make their surfaces hydrophilic. This helps to get the sections flat on the coverslips with fewer wrinkles. To reduce wrinkles of ultrathin sections, the sections were often treated with chloroform.

### Cell culture and virus infection

For cell culture, each coverslip was put in the 6-well, 12-well or 24-well flat-bottom cell culture plates (Corning costar) depending on the coverslip size. BS-C-1 cells (African Green monkey kidney epithelial cells, American Type Culture Collection (ATCC), CCL-26) were cultured in Eagle modified minimum essential medium (ATCC), supplemented with 10% fetal bovine serum (Invitrogen) and antibiotics (ATCC; penicillin and streptomycin). A549 cells (lung carcinoma cells, ATCC CCL-185) were cultured in high glucose Dulbecco’s modified Eagle medium (Invitrogen) supplemented with 10% fetal bovine serum (Invitrogen) and antibiotics (ATCC; penicillin and streptomycin). The cells were maintained in a humidified, 5% CO_2_ environment at 37°C.

For virus infection, Udorn virus strain (a gift from Robert Lamb, Northwestern University, Evanston, IL) was used. A549 cells were infected with a MOI of 3 pfu/cell of A/Udorn/72 for 12 hrs at 37°C and fixed as described below.

### Immunostaining and post-fixation

For imaging viruses budding from cells, Udorn-infected A549 cells were washed in PBS (phosphate-buffered saline) twice after 12 hrs infection, and fixed with a mixture of 4% paraformaldehyde (Electron Microscopy Sciences, 15714) and 0.1% glutaraldehyde (Electron Microscopy Sciences, 16020) at room temperature for 15 minutes (for HA staining or HA and vRNP staining) or fixed with methanol at -20°C for 2min[44]. The cells were then washed with PBS and blocked with 3% (w/v) bovine serum albumin (Jackson ImmunoResearch Laboratories) in PBS for 30 min. For single-color HA staining, cells were incubated with goat HA primary antibodies (NR3118, BEI, Manassas, VA) in blocking buffer for 1hr and Alexa 405 and Alexa 647 co-labeled bovine anti-goat secondary antibodies (Jackson ImmunoResearch Laboratories) for 1hr. For two color imaging, cells were stained with goat HA primary antibodies (or goat M1 primary antibodies (Abcam, ab20910)) and mouse vRNP primary antibodies (Millipore, MAB8800) in blocking buffer for 1hr, followed by Alexa 647 labeled bovine anti-goat secondary antibodies and Alexa 568 labeled anti-mouse secondary antibodies for 1hr. The labeled cells were post-fixed with a mixture of 4% paraformaldehyde and 0.1% glutaraldehyde at room temperature for 10 minutes and washed in PBS. For correlative STORM and BSE-SEM imaging of embedded samples, the post-fixed samples were further fixed and stained with 1% tannic acid (Electron Microscopy Sciences, 21700) in TRIS-maleate buffer (MB, Electron Microscopy Sciences, 11740) pH 6.0 for 10 min, washed with MB for 20 min, and then stained again with 1% uranyl acetate (Electron Microscopy Sciences, 22400) in MB, pH 6.0 for 10 min.

For mitochondria imaging, BS-C-1 cells were washed in PBS twice, fixed with a mixture of 4% paraformaldehyde and 0.1% glutaraldehyde at room temperature for 15 min, and then reduced with 0.1% sodium borohydride for 7 min[38]. After washing with PBS, the cells were permeabilized with 0.2% Triton X-100 and 3% (w/v) bovine serum albumin in PBS for 10 min, and then blocked with 3% (w/v) bovine serum albumin in PBS for 30 min. The cells were stained with rabbit anti-Tom20 (Santa Cruz Biotech, 2 μg/ml) in blocking buffer for 30 min, washed with PBS, and then stained with Alexa 405 and Alexa 647 labeled donkey anti-rabbit secondary antibodies (2 μg/ml) in blocking buffer for 1 hr. The labeled cells were then washed with PBS and post-fixed with 4% paraformaldehyde and 0.1% glutaraldehyde in PBS for 10 min at room temperature. For correlative STORM and BSE-SEM imaging, the post-fixed samples were further fixed and stained with 0.6% osmium tetroxide aqueous solution (Electron Microscopy Sciences, 19152) for 7 min, reduced with 1.5% potassium ferricyanide for 10 min, washed with MB for 20 min, then stained again with 1% uranyl acetate in MB, pH 6.0 for 10 min.

For microtubule staining, BS-C-1 cells were washed in PBS twice, fixed and permeabilized using 0.3% glutaraldehyde and 0.25% Triton X-100 in cytoskeleton buffer (CB: 10 mM MES pH 6.1, 150 mM NaCl, 5 mM EGTA, 5 mM glucose, and 5 mM MgCl_2_) for 5 min in the first step, followed by a second fixation step using 2% glutaraldehyde in CB for 15 min. To reduce the background, the fixed cells were reduced by 0.1% NaBH_4_ in PBS for 7 min[38]. Following washing with PBS for 1 hr, the cells were blocked with 3% (w/v) bovine serum albumin in PBS for 30 min. They were stained for 1hr with rat anti-tubulin primary antibodies (ab6160; Abcam, clone YL1/2) in blocking buffer, washed with PBS, and then stained with Alexa 405 and Alexa 647 co-labeled donkey anti-rat secondary antibodies (2 μg/ml) in blocking buffer for 1 hr. The cells were then washed with PBS and post-fixed with 4% paraformaldehyde and 0.1% glutaraldehyde in PBS for 10 min at room temperature.

### Embedding, sectioning and etching

The fixed and stained cells were washed with double-distilled water three-times for 10 min and then dehydrated in a graded ethanol series (60%, 75%, and 90% ethanol for 5 min each, and followed by 100% ethanol twice for 10 min each). Following dehydration, the sample was infiltrated by progressive incubations with ethanol and UltraBed (a Spurr-like resin, Electron Microscopy Sciences, 14310) at ratios of 2:1, 1:1, and 1:2, followed by 100% resin. After 10 hr infiltration, the sample was embedded and polymerized in 100% UltraBed resin using the BEEM Embedding Capsules (Electron Microscopy Sciences, 69913–05) by baking in a 70°C oven for 17 hrs. For Durcupan ACM embedding, the sample was infiltrated by progressive incubations with ethanol and Durcupan mixture (Electron Microscopy Sciences, 14040), at ratios of 2:1, 1:1, and 1:2, followed by 100% resin. After 10 hr infiltration, the sample was embedded and polymerized in 100% Durcupan resin by heating in a 70°C oven for 17 hrs. For LR White embedding, the sample was infiltrated by progressive incubations with ethanol and LR White (Electron Microscopy Sciences, 14383) at ratios of 2:1, 1:1, and 1:2, followed by 100% resin. To preserve fluorescence, we titrated ethanolamine into LR White to neutralize the resin pH. We chose cold curing by chemical accelerator rather than UV polymerization or heat polymerization to minimize the fluorescence quenching. Following infiltration, the sample was polymerized at -20°C overnight by adding a chemical accelerator (Electron Microscopy Sciences, 14385). The BEEM Embedding Capsules and the plastic coverslip were removed from the hardened resin block by dipping them into liquid nitrogen. The plastic block was trimmed and sectioned with a Leica Ultracut ultramicrotome to obtain the ultrathin sections (70 nm thick). If the section has wrinkles or was not fully stretched, we exposed it to chloroform vapor to make fully stretched section without wrinkles. We placed the sections on a glow-discharged glass coverslips, followed by drying on a hot plate (60°C) for 20 min. The dried sections were etched in 10% saturated sodium ethoxide for 30 sec, washed with double-distilled water and then dried again on a hot plate (60°C) for 10 min.

### STORM imaging

The samples were imaged in an imaging buffer containing 100 mM mercaptoethylamine(MEA) and an oxygen scavernger system [5% glucose (wt/vol), 0.5 mg/ml glucose oxidase (Sigma-Aldrich), and 40 mg/ml catalase (Sigma-Aldrich, C100-50MG)] in PBS at pH 8.5, which has a refractive index of 1.34. For correlative 3D STORM and TEM, higher refractive index (1.45) media was used, which contains 60% (wt/wt) sucrose and 5% (wt/wt) glucose in the imaging buffer described.

When SiN was used as the substrate for the sample, the SIN film was sandwiched between two glass coverslips. The SiN was first placed on a glass coverslip in an imaging buffer, assembled into a sample chamber with another No. 1.5 glass coverslip and the chamber was sealed by nail polish (Electron Microscopy Sciences, 72180) around the edges of a coverslip. When the photo-etched gridded coverslip was used as the substrate for the sample, it was also assembled into a sample chamber with a No. 1.5 glass coverslip with nail polish. The ultrathin sections of embedded samples were placed on one No. 1.5 glass coverslip, assembled into a sample chamber with another No. 1.5 glass coverslips and sealed the same way.

STORM experiments were performed on an Olympus IX71 inverted optical microscope fitted with an Olympus UPlanSApo 100x, 1.4 NA oil immersion, as previously described[27]. Alexa 647 dyes were excited using a 647 nm laser (MPB Comm Inc. 2RU-VFL-P-1500-647) and activated using a 405-nm laser (Cube 405-50C; Coherent). The emission from the Alexa 647 was filtered with a bandpass emission filter (Chroma, ET700/75) and was detected using an electron-multiplying charge-coupled device (EMCCD) camera (Andor Technology, Ixon DU897) at a frame rate of 60 Hz. For 3D STORM imaging, a cylindrical lens with a focal length of 1 m (Thorlabs LJ1836L1-B or LJ1144L1-B) was inserted into the imaging optical path to create astigmatism for 3D imaging, as previously described[27]. For two-color imaging of Alexa 647 and Alexa 568, 561nm and 657nm beams were used to excite Alexa 647 and Alexa 568, respectively, and the dyes were activated by 405nm laser. The emissions from the two dyes were separated by a 630-nm long-pass dichroic mounted on a commercial beamsplitting device (Dual-View; Photometrics), filtered with two bandpass emission filters (FF01-607/70; Semrock and ET705/72m; Chroma) and detected on two different regions of the EMCCD camera at a frame rate of 60 Hz.

### Electron microscopy imaging

After STORM imaging, the substrates with sample still attached were disassembled from the other coverslip by removing the nail polish with ethanol. The samples were washed with double-distilled water before subsequent EM sample preparation steps. All of the following EM sample preparations were performed between STORM and EM imaging.

For correlative 3D STORM and TEM imaging of unembedded sample of microtubules, the sample was incubated in 0.1% aqueous tannic acid for 20 min at room temperature. After washing three times with distilled water for 10 min, it was incubated with 0.1–0.2% uranyl acetate in distilled water for 20 min at room temperature and washed again with distilled water for 10 min. The sample was then dehydrated in a graded ethanol series (60%, 75%, 90% and 100% ethanol for 5 min each, 0.2% uranyl acetate in 100% ethanol for 20min, followed by 100% ethanol twice for 10min each). The samples were further dried with hexamethyldisilazane for 15 min or by Critical Point Drying, and placed on a silicon wafer, followed by sputter-coating with 1 nm of platinum/palladium (Sputter Coater, Cressington, 208HR). For TEM imaging, the samples were imaged in a TecnaiG2 Spirit BioTWIN at 80 kV with an AMT 2k CCD camera.

For correlative 3D STORM and TEM imaging of unembedded samples of mitochondria, the sample was post-fixed with 1% uranyl acetate (Electron Microscopy Sciences, 22400) in distilled water for 10 min at room temperature and washed with distilled water for 10 min. After dehydration in a graded ethanol series (60%, 75%, and 90% ethanol for 5 min each, followed by 100% ethanol twice for 10min each), the samples were further dried with hexamethyldisilazane for 15 min or by Critical Point Drying. The samples were imaged in a TecnaiG2 Spirit BioTWIN at 80 kV with an AMT 2k CCD camera.

For correlative 3D STORM and SEM imaging of unembedded samples, the sample was further fixed in 1% osmium tetroxide aqueous solution (Electron Microscopy Sciences, 19152) for 10 min and then dehydrated in a graded ethanol series (60%, 75%, and 90% ethanol for 5 min each, and followed by 100% ethanol twice for 10 min each). The samples were further dried with Hexamethyldisilazane (HMDS, Electron Microscopy Sciences, 16782) for 15 min or by Critical Point Drying (CPD, Tousimis, Auto Samdri 815 Series A), and placed on a silicon wafer followed by carbon-coating with the carbon evaporator (HHV Auto306). The samples were imaged using an Ultra55 Field Emission Scanning Electron Microscope (FESEM, Zeiss) at 3 keV after identifying the same regions that were imaged with STORM by reading the number and grid on the photo-etched gridded coverslip.

For correlative STORM and BSE-SEM imaging of embedded samples, the sample sections were further stained by 2% uranyl acetate in ethanol solution for 10 min at room temperature, washed three times with distilled water, and then stained with Reynold’s lead citrate for 5 min at room temperature. The stained sections were rinsed again with distilled water and dried overnight. The coverslips with the sections were placed on a silicon wafer, and an additional carbon layer was sputtered on the sections for conductivity by using a carbon evaporator (HHV Auto306). The samples were imaged in a Zeiss Sigma Field Emission Scanning Electron Microscope (FESEM, Zeiss) with a the accelerating voltage of 7 keV by detecting back-scattered electrons and inverting black/white signal for easier visualization of the structure.

### Image analysis

Before overlaying STORM and EM images, the brightness and contrast of the EM images was adjusted using PHOTOSHOP (Adobe Systems, San Jose, CA, U.S.A.) and the EM image was roughly rescaled until the scale bar matched the size of the scale bar on the STORM image. As a first rough alignment, the low magnification STORM image and the corresponding EM image were overlaid by rescaling, translation and rotating using PHOTOSHOP or the image registration function Control Point Registration in Matlab (The Math Works Inc., Natick, MA). Features visualized in both images could be used as fiduciary markers in this step, including gold fiduciary markers, the silicon nitride support mesh in the SiN window, dirt, the edge of the section and wrinkles in the section.

After the rough alignment, a refined alignment between two images was performed based on structures within the cells, such as virus filaments, mitochondria, and microtubules, by rescaling, translation and rotating the images using PHOTOSHOP and Matlab. In order to estimate how accurate the correlation is, we calculated the normalized cross-correlation between STORM and EM images. To compute the normalized cross-correlation between STORM and EM images, images were converted to grayscale images and the cross-correlation between the EM and STOMR images was calculated using the 'normxcorr2' function of Matlab and displayed as a surface plot.

### Photon number and number of switching cycles analysis

To investigate the effect of sample preparation steps on the photon output of the dyes, we analyzed the average photon number associated with more than 2,000,000 detected molecular localizations (each corresponding to a switching event of the dye molecules in the field of view) for each condition. We also calculated the mean values from the single exponential fit of the photon number distributions and obtained similar results to the average values. The results were normalized against the control condition, in which sample was post-fixed only with 4% PFA and 0.1% GA after immunolabeling without any additional fixation (with osmium, tannic acid or uranyl acetate), dehydration, polymerization, sectioning or etching. In order to investigate the effect of sample preparation steps on the number of switching cycles per fluorophore, we analyzed more than 1,000 detected dye molecules for each condition. From the fluorescence time traces of individual dye molecules, switching events were identified and counted.
